# Clinical Outcomes of Total or Partial Renal Artery Embolization in Patients with Spontaneous Renal Bleeding

**DOI:** 10.2174/0115734056355268241230071424

**Published:** 2025-01-03

**Authors:** Hyo Jeong Lee, Chang Hoon Oh, Soo Buem Cho, Sang Lim Choi

**Affiliations:** 1 Department of Radiology, College of Medicine, Ewha Womans University Mokdong Hospital, Seoul, Republic of Korea; 2 Department of Radiology, College of Medicine, Ewha Womans University Seoul Hospital, Seoul, Republic of Korea; 3 Department of Radiology, Chung-Ang University Gwangmyeong Hospital, Gwangmyeong, Republic of Korea

**Keywords:** Embolization, Renal artery, Spontaneous bleeding, Endovascular treatment, n-butyl cyanoacrylate, Microcoil

## Abstract

**Aims:**

The aim of this study was to evaluate renal artery embolization in patients with spontaneous renal artery bleeding based on detailed angiographic findings and a comprehensive analysis of its efficacy and clinical outcomes.

**Materials and Methods:**

This retrospective study evaluated the outcomes of renal artery embolization in 18 cases among 15 patients (11 men and 4 women; mean age: 57.9 years) treated for spontaneous renal bleeding at our institution between March 2017 and October 2023. Data derived from abdominal computed tomography (CT) and arteriography were analyzed to assess the effectiveness of embolization.

**Results:**

Most patients had end-stage renal disease or renal atrophy, with common findings on CT scans, including signs of active bleeding in 66.7% (10/15) and hematoma extending to the retroperitoneal space in 53.3% (8/15). Microcoils were commonly used for embolization (*n* = 10), with a technical success rate of 100% and primary and final clinical success rates of 80% and 100%, respectively. No major complications were reported during the follow-up, and clinical improvement was observed in all patients who underwent total embolization, with few instances of reduced hematoma size and renal atrophy.

**Conclusion:**

Transarterial embolization is safe and effective for controlling spontaneous renal hemorrhage.

## 1 INTRODUCTION

Renal artery bleeding commonly occurs secondary to blunt trauma or iatrogenic injuries, such as percutaneous renal biopsy, laparoscopic or robotic partial nephrectomy, percutaneous nephrostomy tube placement, and percutaneous nephrostolithotomy, and it can be potentially fatal. However, most cases of renal artery bleeding are self-limiting and do not require intervention [1-4]. Spontaneous renal hemorrhage is a rare condition that is frequently associated with acquired cystic kidney disease (ACKD), anticoagulation medication, or bleeding diathesis. However, it can also be observed in patients with end-stage renal disease (ESRD) and a long history of hemodialysis, arteriosclerosis, or arteritis [5, 6].

In most patients, the bleeding site is inconspicuous and is usually secondary to capillary lesions. Treatment is generally conservative treatment, including correction of coagulopathy and blood transfusion or open surgical procedures such as partial or total nephrectomy or arterial ligation in patients with hemodynamic instability [7]. However, transcatheter arterial embolization is now considered the most appropriate technique owing to its less invasive nature and high success rate in patients with poor hemodynamic tolerance [8, 9].

Existing studies on renal artery embolization for spontaneous renal artery hemorrhage are scarce; therefore, this retrospective study aimed to evaluate the efficacy and clinical outcomes of renal artery embolization in patients with spontaneous renal artery bleeding based on detailed angiographic findings.

## 2 MATERIALS AND METHODS

### 2.1 Patients

This retrospective single-center study was approved by the institutional review board of Ewha Womans University (IRB No. EUMC 2024-02-025), which waived the need for obtaining informed consent from the patients. This study enrolled patients who had undergone renal artery embolization at our hospital between March 2017 and October 2023. All methods were performed in accordance with the relevant guidelines and regulations. Patients with spontaneous hemorrhage were included, and those with obvious renal tumors or traumatic renal injuries were excluded. The final analysis included 15 patients (11 men and 4 women), with a mean age of 57.9 years (age range, 45–94 years), who had undergone renal artery embolization for spontaneous renal bleeding. All radiographic images, including abdominal computed tomography (CT) scans and arteriograms, were collected using our imaging archiving system. The decision to perform renal angiography and subsequent embolization was made for patients in whom renal hemorrhage was confirmed by CT. However, even in cases where CT did not reveal active bleeding, the procedure was performed if clinical and laboratory findings suggested continuous bleeding.

### 2.2 Angiography and Embolization Technique

Renal artery embolization was performed by five board-certified interventional radiologists with 5–15 years of clinical experience. Under local anesthesia, the right common femoral artery was accessed, and a 5-F Cobra catheter (Cobra catheter; Cook, Bloomington, IN, USA) was advanced over a 0.035-inch hydrophilic guidewire (Radifocus; Terumo, Tokyo, Japan) for selective renal artery catheterization, and renal angiography was performed. The presence of accessory renal arteries should be carefully evaluated using preoperative CT or MRI or during angiography to avoid incomplete embolization. Based on the location of the target lesion, branching arteries were selectively catheterized using a coaxial 1.7–1.9-Fr microcatheter. During renal angiography, contrast medium was administered at a flow rate of 2–3 mL/s, with a total volume of approximately 4–6 mL. For angiography at the interlobar artery or lower levels using a microcatheter, the contrast medium was delivered at a flow rate of 0.5–1 mL/s, with a total volume of 1.5–3 mL. Embolic agents included polyvinyl alcohol (PVA; Contour; Boston Scientific, Marlborough, MA, USA), n-butyl cyanoacrylate (NBCA; Histoacryl; B. Braun, Melsungen, Germany), and lipiodol (Guerbet, Villepinte, France) mixture in a ratio of 1:3, detachable vascular micro coils (Interlock; Boston Scientific, Marlborough, MA, USA, and Concerto; Medtronic, Minneapolis, MN, USA), and gelatin sponge particles (GSP; Nexsphere, Next Biomedical, Incheon, Korea) or their combinations. The choice of embolic agent was entrusted to the attending physician’s discretion [10, 11].

### 2.3 Study Endpoints

Technical success was defined as the complete hemostasis of the bleeding vessel and the absence of any visible signs of active bleeding, such as contrast medium extravasation (CME), on the immediate post-embolization angiogram. Primary clinical success was defined as the resolution of bleeding symptoms without the need for additional interventional or surgical hemostasis following the initial session. Final clinical success was defined as the resolution of signs and symptoms of bleeding with no further intervention, regardless of the number of procedures performed. Partial embolization was defined as the superselective embolization of the renal artery's branches, specifically the segmental renal artery, interlobar artery, or arcuate artery, rather than targeting the main renal artery and the presence of remaining arterial flow to the kidney after embolization. Total embolization was defined as embolizing the main renal artery and a complete absence of arterial flow to the kidney, as evidenced by post-embolization angiography. Complications were classified as major or minor according to the Society of Interventional Radiology Standards of Practice Committee guidelines [12]. Minor complications were defined as those requiring no additional treatment or overnight hospitalization for observation. Major complications were defined as those requiring intervention with hospitalization < 48 h, need for major intervention, prolonged hospitalization (> 48 h), unanticipated increase in the level of care, permanent adverse sequelae, or death. Among these complications, contrast-induced nephropathy (CIN) was defined as a 25% increase in the serum creatinine level from baseline within 48–72 h of administration of the contrast agent without apparent causes [13].

## 3 RESULTS

Table **1** summarizes the patients’ clinical characteristics and laboratory and CT findings. Pain in the abdominal or flank area on the affected side was reported in 14 patients (93.3%), and one patient (6.7%, patient no. 7) presented with general weakness to the emergency department of our institution. Hemodynamic instability was initially reported in three patients (20%). Of the 15 included patients, CT findings were indicative of ESRD in 10; among them, 7 had ACKD. All patients with ESRD received hemodialysis at our institution, and low-molecular-weight heparin was used for heparinization. Among them, one patient (patient no. 9) with ESRD and ACKD had a history of cerebral infarction and was on oral anticoagulant therapy. Additionally, two patients without ESRD had coronary artery occlusive disease (CAOD) and were on oral anticoagulant therapy. One patient (patient no. 15) had underlying colon cancer with atrophic changes in the kidney on the side where the CT showed hemorrhage, although they had never been diagnosed ESRD, while the remaining two patients had no pertinent medical history or oral medications. There were no patients with coagulopathy in our study.

CT scans were performed in all patients, which revealed signs of active bleeding such as CME or pseudoaneurysm in 10 patients (66.7%), confined hematoma including subcapsular hematoma (SCH) or perirenal hematoma (PRH) in 7 patients (46.7%), and hematoma extending beyond the perirenal fascia into the retroperitoneal space in 8 patients (53.3%). Among them, four patients (26.7%) showed non-delineated renal parenchyma, indicative of a scattered kidney. Of the seven patients with ESRD and ACKD, CT findings revealed hemorrhagic cyst rupture due to high-density lesions within the cysts in two patients (28.6%). Although bleeding occurred in an unrelated area, renal cell carcinoma was incidentally diagnosed in one patient. The mean platelet count was 162.5 x 10^3^/μL (range: 102–256 x 10^3^/μL), and the mean activated partial thromboplastin time was 25.7 s (range: 19.0–34.6 s).

Table **2** summarizes the overall details of TAE in patients. Angiography revealed findings suggestive of CME in 10 patients, indicating active bleeding and pseudoaneurysm in 3 patients. Two individuals did not show clear signs of active bleeding. Among the 13 patients with bleeding, nine (69.3%) had bleeding from multiple renal artery branches. Additionally, among these, a hematoma extending beyond the perirenal fascia into the retroperitoneal space was observed on CT in eight patients. The eight patients on dialysis for ESRD and one patient (patient no. 5) with elevated baseline creatinine (2.66mg/dL) showed decreased renal parenchymal staining (RPS), refers to the hyperattenuation observed in the kidney tissue following the administration of contrast media on angiography. The embolic materials used were as follows: microcoils+GSP (*n* = 4), NBCA+PVA (*n* = 4), microcoils alone (*n* = 3), microcoils+PVA (*n* = 2), microcoils+NBCA (*n* = 1), and NBCA alone (*n* = 1). Total and partial embolizations were performed in 8 (53.3%) and 7 (46.7%) patients, respectively. CME or pseudoaneurysm was reported in six of the eight patients (75.0%) who underwent total embolization, indicative of active bleeding on angiography. Prophylactic total renal artery embolization was performed using only coils in two patients in whom active bleeding was not clearly visible on angiography. A combination of two or more types of embolic materials was used for 11 of the 15 patients. In patients who underwent total embolization (*n* = 8), reflux of contrast media to the aorta due to resistance (RCMR), which refers to a condition during angiography where the flow of contrast media to the renal artery and its branches is less prominent compared to the reflux back to the aorta and decreased RPS were reported in 8 and 7 patients, respectively (Table **2**).

**Fig. (1):**
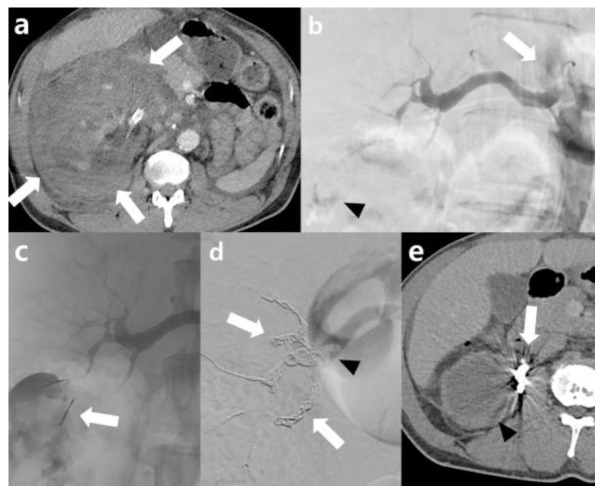
48-Year-old male patient with ESRD. (**a**) Computed tomography (CT) showed huge retroperitoneal hematoma (arrow) with contrast media extravasation and non-delinated renal parenchyma of right kidney. (**b**) Selective right renal artery angiograms showing reflux of contrast media to the aorta due to resistance (arrow), contrast media extravasation (arrowhead), and decreased renal parenchymal staining. (**c**) Embolization was performed using microcoils (arrow) and n-butyl cyanoacrylate (NBCA) at interlobar arteries and subsequent post-embolization angiography did not show signs of active bleeding. (**d**) After 6 hours owing to persistent vital sign instabilities and a decrease in hemoglobin, total renal artery embolization was performed using microcoils (arrow) and NBCA (arrowhead) at segmental arteries to the main renal artery. (**e**) Follow-up CT showed atrophic changes and absence of enhancement in the right kidney, with a decrease in the size of the hematoma and liquefaction.

The technical, primary, and final clinical success rates were 100%, 80% (12 / 15), and 100%, respectively (Table **3**). One patient (patient no. 6) was excluded from the evaluation of final clinical success due to the impossibility of assessment caused by a subsequent nephrectomy on the same day. Partial renal arterial embolization was initially performed in two patients (patients no. 4 and 12) who experienced primary clinical failure with evidence of multifocal CME and decreased RPS and RCMR on the first angiography. However, owing to their poor clinical condition, both patients underwent total renal arterial embolization on the second angiography. In one patient (patient no. 4), initial abdominal aortography did not show arterial flow to the right renal artery. Subsequent right renal angiography revealed RCMR, decreased PRSRPS, and multifocal active bleeding; therefore, embolization of the interlobar arteries was performed using micro coils and NBCA. Although post-embolization angiography did not show signs of CME or active bleeding, transarterial embolization (TAE) was performed after 6 h owing to persistent vital sign instabilities and a decrease in hemoglobin. Multiple segmental arteries to the main renal artery were embolized using micro-coils and NBCA. A follow-up CT performed after 4 months showed atrophic changes and absence of enhancement in the right kidney, with a decrease in the size of the hematoma and liquefaction (Fig. **1**).

The overall mean follow-up period for all patients was 12.1 months (range: 1–48 months), except for two patients who underwent surgical nephrectomy 6 h and 2 days after TAE. Clinical improvement was observed in all eight patients who underwent total renal artery embolization. Among them, two patients did not undergo follow-up imaging due to follow-up loss. In the remaining six patients, a notable reduction in hematoma size and interval renal atrophy were observed. Renal atrophy and no renal enhancement were observed in four patients; however, two patients (patients no. 10 and 14), in whom only micro coils were used for prophylactic embolization, exhibited partial renal enhancement with interval decreased kidney size on follow-up CT scans. No major complications were reported in any patient. Two patients (patients 1 and 8) experienced post-embolization syndrome, characterized by mild abdominal pain and fever within 24 h after embolization, which subsided after 48 h, with no signs of infection. Contrast-induced nephropathy was reported in three patients (patients 8, 9, and 13), which stabilized following hemodialysis at 2–3 days postoperatively.

## 4 DISCUSSION

To minimize post-embolization syndrome and preserve optimal renal function, super-selective embolization should be performed to target the bleeding site [1, 14]. Nevertheless, total renal artery embolization is performed in a few instances, such as in patients with ESRD, irreversible transplant rejection, arteriovenous fistula, ectopic kidney with urinary incontinence [15-22], traumatic renal injuries (Grade IV), according to the American Association for the Surgery of Trauma renal injury grading system [23], or iatrogenic ureteral injuries. Moreover, in patients with ACKD, previous studies have indicated that total embolization for spontaneous renal hemorrhage enhances technical success without impairing urine production [24]. In our study, the technical success rate was 100%, which is consistent with those of previous studies ranging from 80% to 100% [24-26]. Clinical improvement was achieved in 80% of patients after a single-session TAE. However, excluding two patients who underwent nephrectomy within hours to days after the procedure—making it impossible to assess final clinical success—the remaining patients demonstrated a 100% final clinical success rate with no major complications. Notably, among the eight patients who underwent total embolization, all achieved favorable outcomes. These findings are consistent with a previous study on spontaneous renal hemorrhage in patients with ACKD, which reported a 100% technical success rate and a 94% clinical success rate [24].

Angiography revealed a decreased RPS in 10 patients (66.7%), including nine ESRD patients, and one had an elevated serum Cr of 2.66 mg/dL and had not undergone dialysis. In our study, among the 10 patients with decreased RPS, eight exhibited bleeding from multiple renal artery branches on angiography. Total embolization was performed in seven patients, excluding one patient for whom selection was relatively easy. Super-selective embolization was initially performed for two patients to minimize damage. However, their condition deteriorated, necessitating total renal artery embolization in the second session, which subsequently led to improvement. This finding may be partly attributed to the tendency of ESRD patients to have small, irregular renal arteries that can narrow under shock conditions, potentially leading to spasms [25], which could explain the observed decrease in RPS on angiography. The difficulty in performing superselective embolization of the bleeding artery to minimize renal parenchymal damage is the main cause of technical failures [25, 26]. Because arterial diameters constrict under shock conditions and can react spastically to the catheter or guidewire manipulation, performing super-selective embolization can be challenging [25].

In our study, patients without ESRD exhibited less extensive spontaneous renal bleeding compared to those with ESRD or renal atrophy. Specifically, four non-ESRD patients experienced bleeding that was more confined to the perirenal space, presenting as SCH or PRH on CT. Among them, two patients with SCH had no underlying disease or medication history, while the other two, who had relatively more extensive hematomas like PRH, were on anticoagulants for CAOD. Consequently, super-selective embolization was performed in these four patients to preserve renal function as much as possible. In contrast, 11 ESRD patients undergoing hemodialysis or with renal atrophy experienced more extensive hemorrhage, often involving hematoma extension beyond the perirenal fascia into the retroperitoneal space. This difference may be attributed to cortical atrophy and scarring in ESRD patients, which lead to capsular detachment and tearing of the cortical arteries, exacerbating the severity of bleeding. Capsular perforation further diminishes the tamponading effect of subcapsular hematomas, allowing blood to leak into surrounding spaces. On CT, this often presents as a perirenal hematoma or one extending beyond the perirenal fascia into the retroperitoneal space [27]. The rarity of spontaneous renal bleeding as a condition, combined with the varying extent of hemorrhage between patient groups, highlights the need for further research with larger cohorts to make more accurate comparisons.

Prophylactic embolization has proven to be an effective approach, particularly in patients with extensive hemorrhage. In our study, eight patients (53.3%) with hematoma extending to the retroperitoneal space underwent total embolization, including two patients (25%) who showed no definite active bleeding on angiography. Despite the absence of visible bleeding, prophylactic total embolization of the main renal artery was performed in these two patients using micro coils to prevent reflux of embolic material into the aorta, a complication associated with reduced flow in small renal arteries commonly observed in ESRD or chronically diseased kidneys [19]. The procedure was completed after confirming the absence of blood flow on post-embolization angiography, and both patients showed clinical improvement without any significant complications, consistent with findings from a previous study reporting a 94% clinical success rate following prophylactic embolization [24]. Furthermore, proximal embolization of the main renal artery using microcoils minimizes the risk of complete renal necrosis or infarction secondary to capillary embolization [28]. These findings emphasize that prophylactic embolization can be a quick and efficient strategy to prevent clinical failures and achieve favorable outcomes, especially in patients with ESRD or poor renal function who present with decreased RPS, multiple hemorrhages on angiography, or hematoma extending beyond the perirenal fascia into the retroperitoneal space on CT.

Necrotic pyelonephritis of the infarcted kidney is a major complication of renal artery embolization [29]. However, no major complications were reported in our study, and only 13.3% (2 / 15) of patients experienced minor complications, such as post-embolization syndrome, characterized by fever and abdominal pain, which improved within a day or two with conservative treatment. The lower frequency of complications related to renal infarction could be attributed to total embolization performed on patients with ESRD or atrophied kidneys than in those with normal kidney function. Generally, the likelihood of infarction is less in patients with ESRD because of the small volume of renal parenchyma [29]. Nevertheless, 20% (3 of 15 patients) experienced CIN, all of whom had ESRD. Among these, only one patient (patient no. 9) underwent total embolization, warranting further research on the relationship between total embolization and CIN. Serum creatinine elevation in these cases could result from both CIN and transient renal blood flow reduction due to embolization [24]. Although our study did not clearly differentiate these mechanisms, adequate hydration and minimal contrast use were employed to mitigate CIN risk. The absence of long-term renal dysfunction suggests the observed changes were likely temporary. Further research is needed to distinguish CIN from embolization-related renal impairment.

Total arterial embolization can also be performed for spleen injuries within the visceral arteries. Proximal splenic artery embolization (PSAE) is indicated in cases of multifocal injury or when no focal angiographic abnormality is found, but CT scans show evidence of injury [30]. PSAE reduces the spleen's systolic arterial pressure, promoting hemostasis and healing, while collateral blood flow maintains spleen function and prevents infarction abscess formation, and preserves immune function [30-32]. PSAE is preferred over distal embolization for its speed and simplicity, especially beneficial for patients with challenging anatomy or conditions such as vasospasm or vascular disease [30, 33]. A recent study by Boscà-Ramon, A., *et al*. showed an overall high technical (100%) and clinical (94.5%) success, as well as a low overall complications rate of 5.1% [34]. A recent systematic review reported a similar overall success rate of 90% and a major complication rate of 6.4%, similar to our study's outcomes of 100% technical and final clinical success, with no major complications [33]. Notably, in our study, out of the eight patients who underwent total embolization, seven were diagnosed with ESRD, and one exhibited renal atrophy, essentially indicating patients with minimally functioning kidneys. This suggests that despite the kidneys being end arteries with almost no collateral pathways, unlike the spleen, we were able to achieve high success rates without encountering complications.

Our study had certain limitations. The smaller sample size and the retrospective nature of the study are major limitations. Studies with larger patient cohorts and longer follow-up periods are required to validate the safety and efficacy of TAE for spontaneous renal hemorrhage. However, this can be explained by the rarity of spontaneous renal hemorrhage. Second, the choice of embolic agents and devices was based on operator preference, making it challenging to assess the impact of embolic agents on renal artery embolization. Third, routine follow-up with contrast-enhanced CT was not performed, resulting in a loss of follow-up or varying timing of CT scans among the patients. Finally, the present study did not have a comparator cohort consisting of patients managed through surgical intervention or conservative treatment. Due to this limitation, it is challenging to ascertain the relative efficacy of embolization against these alternative therapeutic strategies. In future studies, it would be important to include such a comparison to get a clearer understanding of the best ways to treat patients.

## CONCLUSION

Renal artery embolization is safe and effective for spontaneous renal hemorrhage. Furthermore, total renal artery embolization could be effective in ESRD patients with CT findings indicative of hematoma extending to the retroperitoneal space or multiple bleeding events and decreased RPS observed on angiography.

## Figures and Tables

**Table 1:** Patients characteristics, computed tomography finding, and baseline laboratory finding.

Patient No.	Age/Sex	Underlying Disease	Hemodialysis	Oral Anticoagulation	Clinical Manifestation	Direction	Computed Tomography Finding	Baseline Laboratory Finding			
								Platelet (10^3^/μL)	INR	Creatine (mg/dL)	BUN (mg/dL)
1	M/73	CAOD	No	Yes	Right abdominal pain	Right	PRH + CME	141	1.06	1.27	18
2	M/45	None	No	No	Flank pain	Right	SCH + CME	256	1.12	1.41	18
3	F/63	None	No	No	Flank pain	Right	SCH	248	1.06	0.70	10
4	M/48	ESRD	Yes	No	Flank pain	Right	PRH extending retroperitoneum + CME + Non-delinated renal parenchyma	117	1.05	7.64	37
5	F/94	HTN, CAOD	No	Yes	Flank pain	Left	PRH + CME	186	1.44	2.66	34
6	M/75	ESRD with ACKD, HTN	Yes	No	Flank pain	Right	SCH + CME	123	1.08	6.83	33
7	F/76	ESRD, HTN, DM	Yes	No	General weakness	Right	PRH extending retroperitoneum	216	0.96	3.43	16
8	M/46	ESRD with ACKD	Yes	No	Flank pain, vomiting	Right	PRH + high density lesion within cyst, R/O hemorrhagic cyst rupture	243	0.93	12.4	60
9	M/60	ESRD with ACKD, HTN, DM, Cerebral infarction	Yes	Yes	Flank pain	Right	PRH extending retroperitoneum + CME	112	1.22	5.77	47
10	F/52	ESRD with ACKD	Yes	No	Flank pain	Left	PRH extending retroperitoneum + high density lesion within cyst, R/O hemorrhagic cyst rupture	134	0.94	11.32	68
11	M/63	ESRD with ACKD	Yes	No	Flank pain	Right	PRH extending retroperitoneum + CME + Non-delinated renal parenchyma	142	1.20	7.53	49
12	M/48	ESRD with ACKD	Yes	No	Flank pain	Right	PRH extending retroperitoneum + CME	102	1.25	9.09	46
13	M/45	ESRD with ACKD, Renal cell carcinoma	Yes	No	Flank pain	Right	PRH + CME	159	1.01	11.64	66
14	M/53	ESRD	Yes	No	Flank pain	Left	PRH extending retroperitoneum + CME + Non-delinated renal parenchyma	107	1.34	10.92	61
15	M/61	Colon cancer, left kidney atrophy	No	No	Flank pain	Left	PRH extending retroperitoneum	152	1.15	0.98	19

Note: * CAOD coronary artery occlusive disease, ESRD end stage renal disease, HTN hypertension, ACKD acquired cystic kidney disease, DM diabetes millitus, SCH subcapsular hematoma, PRH perirenal hematoma, CME contrast media extravasation, INR international normalized ratio, BUN blood urea nitrogen.

**Table 2:** Intervention details, angiographic findings, and dmbolization characteristics.

Patient No.	Session	Multiple Bleeding	Angiography Finding	Embolic Agent	Embolized Artery	Hemoglobin (g/dL, Pre / Post)
1	1	Yes	CME	Microcoils + GSP	Actuate artery	10.8 / 13.0
2	2	Yes	CME	Microcoils + GSP	Interlobar artery → interlobar artery	12.1 → 8.5 → 10.8
3	1	No	Pseudoaneurysm	Microcoils + GSP	Actuate artery	9.9 / 10.5
4	2	Yes	CME + RCMR + Decreased RPS	Microcoils + NBCA	Interlobar artery → main renal artery	6.2 → 5.9 → 9.2
5	1	No	CME + RCMR + Decreased RPS	Microcoils	Segmental artery	7.9 / 8.6
6	1	No	CME	Microcoils + GSP	Actuate artery	7.4 / 9.0
7	1	Yes	Pseudoaneurysm + RCMR + Decreased RPS	NBCA + PVA	Main renal artery	5.4 / 8.5
8	1	No	CME+ RCMR + Decreased RPS	NBCA	Interlobar artery	8.9 / 10.7
9	1	Yes	CME + RCMR + Decreased RPS	NBCA + PVA	Main renal artery	8.1 / 9.4
10	1	No	RCMR + Decreased RPS	Microcoils	Main renal artery	5.2 / 10.6
11	1	Yes	Pseudoaneurysm + RCMR + Decreased RPS	Microcoils + PVA	Main renal artery	8.5 / 9.2
12	2	Yes	CME + RCMR + Decreased RPS	NBCA + PVA	Interlobar artery → main renal artery	7.2 → 7.4 → 9.1
13	1	Yes	CME + RCMR+ Decreased RPS	NBCA + PVA	Interlobar artery	7.9 / 9.1
14	1	No	RCMR + Decreased RPS	Microcoils	Main renal artery	6.2 / 8.3
15	1	Yes	CME + RCMR	Microcoils + PVA	Main renal artery	8.2 / 9.4

Note: * CME contrast media extravasation, RCMR reflux of contrast media to the aorta due to resistance, RPS renal parenchymal staining, PVA polyvinyl alcohol, GSP gelatin sponge particles, NBCA n-butyl cyanoacrylate.

**Table 3:** Outcomes of interventions including technical and clinical success, complications, and follow-up findings.

Patient No.	Type of Embolization	Technical Success	Clinical Success	Complication	Follow-up (months)	Remarks
			Primary / Final	Major / Minor		
1	Partial	Yes	Yes / Yes	None / fever & abdominal pain	4	Almost disappeared hematoma on CT
2	Partial → Partial	Yes	No / Yes	None / None	5	Continuous hemodynamic instability → 2^nd^ Transarterial embolization at another interlobar artery using GSP and microcoils
3	Partial	Yes	Yes / Yes	None / None	4	Almost disappeared hematoma on CT
4	Complete	Yes	No / Yes	None / None	4	Continuous hemodynamic instability → 2^nd^ Transarterial embolization at main renal artery using microcoils and NBCA 4 month F-U: Atrophy and no enhancement of kidney on CT
5	Partial	Yes	Yes / Yes	None / None	2	Follow-up loss after 2 months
6	Partial	Yes	Yes / -	None / None	-	Vital sign stable after embolization → Surgical nephrectomy at the same day (impossible evaluation of final clinical success due to subsequent nephrectomy on same day)
7	Complete	Yes	Yes / Yes	None / None	18	Almost disappeared hematoma on CT 18 month F-U: Atrophy and no enhancement of kidney on CT
8	Partial	Yes	Yes / Yes	None / fever & abdominal pain + CIN	1	Decreased amount of hematoma and no active bleeding on CT and follow-up loss CIN → improved after 2days hemodialysis
9	Complete	Yes	Yes / Yes	None / CIN	31	31 month F-U: Atrophy and no enhancement of kidney on CT CIN → improved after 3days hemodialysis
10	Complete	Yes	Yes / Yes	None / None	8	1 month F-U: Interval atrophy, but reisudal residual enhancement of kidney on CT
11	Complete	Yes	Yes / Yes	None / None	15	Clinical improvement, but no image follow-up
12	Partial → Complete	Yes	No / Yes	None / None	48	Continuous hemodynamic instability → 2^nd^ Transarterial embolization at main renal artery using NBCA 48 month F-U: Atrophy and no enhancement of kidney
13	Partial	Yes	Yes / Yes -	None / CIN	-	Radical nephrectomy for renal cell carcinoma after 2 days CIN → improved after 2days hemodialysis
14	Complete	Yes	Yes / Yes	None / None	15	2 month F-U: Interval atrophy, but reisudal residual enhancement of kidney on CT
15	Complete	Yes	Yes / Yes	None / None	2	Clinical improvement, but no image follow-up

Note: * CIN contrast induced nephropathy, CT computed tomography.

## Data Availability

The data and supportive information are available within the article.

## LIST OF ABBREVIATIONS

ACKD: Acquired Cystic Kidney Disease
ESRD: End-stage Renal Disease
CT: Computed Tomography
CME: Contrast Medium Extravasation
CIN: Contrast-Induced Nephropathy
CAOD: Coronary Artery Occlusive Disease
TAE: Transarterial Embolization
PSAE: Proximal Splenic Artery Embolization

## STANDARDS OF REPORTING

STROBE guidelines were followed.
